# Preparation and Properties of Bimetallic Chitosan Spherical Microgels

**DOI:** 10.3390/polym15061480

**Published:** 2023-03-16

**Authors:** Andrea Lončarević, Karla Ostojić, Inga Urlić, Anamarija Rogina

**Affiliations:** 1 Faculty of Chemical Engineering and Technology, University of Zagreb, Trg Marka Marulića 19, HR-10000 Zagreb, Croatia; 2 Faculty of Science, University of Zagreb, Horvatovac 102a, HR-10000 Zagreb, Croatia

**Keywords:** bimetallic microgel, electrospraying, copper, zinc, chitosan, cytotoxicity

## Abstract

The aim of this work was to prepare bimetallic chitosan microgels with high sphericity and investigate the influences of metal-ion type and content on the size, morphology, swelling, degradation and biological properties of microgels. Amino and hydroxyl groups of chitosan (deacetylation degree, *DD*, of 83.2% and 96.9%) served as ligands in the Cu^2+^–Zn^2+^/chitosan complexes with various contents of cupric and zinc ions. The electrohydrodynamic atomization process was used to produce highly spherical microgels with a narrow size distribution and with surface morphology changing from wrinkled to smooth by increasing Cu^2+^ ions’ quantity in bimetallic systems for both used chitosans. The size of the bimetallic chitosan particles was estimated to be between 60 and 110 µm for both used chitosans, and FTIR spectroscopy indicated the formation of complexes through physical interactions between the chitosans’ functional groups and metal ions. The swelling capacity of bimetallic chitosan particles decreases as the *DD* and copper (II) ion content increase as a result of stronger complexation with respect to zinc (II) ions. Bimetallic chitosan microgels showed good stability during four weeks of enzymatic degradation, and bimetallic systems with smaller amounts of Cu^2+^ ions showed good cytocompatibility for both used chitosans.

## 1. Introduction

Tissue regeneration in small defects could be assisted by implanted biomaterials that mimic the properties of a tissue-specific extracellular matrix. Among the different types of biomaterials, hydrogels can serve as a good platform for tissue regeneration due to their large surface-to-volume ratio and high porosity. Furthermore, their high water content allows a microenvironment that can mimic a tissue’s architecture to direct cell organization [1]. Synthetic and natural polymers were used to prepare hydrogels systems for specific applications with an emphasis on natural polymers regarding their biocompatibility. Collagen, alginate, gelatin and chitosan-based hydrogels were extensively investigated and developed as temporary cell supports or cell carriers for soft and hard tissue regeneration [2]. A hydrogel’s three-dimensional network allows for the encapsulation of biomolecules, leading to hydrogel’s functionalization for tissue engineering and drug delivery applications. The ability to be physically crosslinked through weak bonds makes hydrogels promising systems for the delivery of biomolecules by preserving their structural integrity [3].

Recently, natural polymer-based hydrogels containing bioactive ions (such as Cu, Zn, Ga, B, Mn and Fe ions) have provided alternatives to biomolecule-functionalized hydrogel biomaterials. The usage of so-called therapeutic ions can induce specific biological functions, such as osteogenesis, angiogenesis and antibacterial activity [4]. On the other hand, the appropriate amount of metal ions could assist in chemotherapies [5]. For instance, injectable alginate hydrogel functionalized by borax, as a source of borate ions, promoted muscle regeneration after injury [6]. Furthermore, the proposed hydrogel has indicated potential as an ion-delivery system for specific stimulation of cell integrins. The physicochemical properties of alginate hydrogels can be improved by a simple mixing in of two metal ions. The synergistic effect of divalent and multivalent ions (Fe^3+^, Cu^2+^, Ni^2+^, Mn^2+^, Co^2+^) was observed by improved mechanical, swelling and self-healing properties of a bimetallic alginate-based hydrogel [7]. Another study on Zn/Cu–gelatin hydrogels [8] focused on synchronous regeneration of gradient tissue injury. Aside from zinc and copper’s role as gelatin crosslinkers, the hydrogels promoted tenogenesis and osteogenesis in vivo, and possess good antibacterial ability. 

The functionalization of chitosan-based hydrogels by divalent and multivalent metal ions has been substantially investigated in the last decade. Chitosan is a natural polycation consisted of *N*-acetyl D-glucosamine and D-glucosamine units and possesses a structure similar to that of ECM components. Chitosan’s functional groups allow for metal-ion complexation at specific concentrations of metal ions, resulting in hydrogels with enhanced antibacterial properties or cytotoxic activity [9,10,11]. The biocompatibility of copper-functionalized chitosan-based hydrogels can be modulated by using a lower copper-ion concentration, while improving their antibacterial and antimicrobial properties [12,13]. On the other hand, chitosan–zinc complexes showed potential for wound healing through improved cell viability, angiogenic behavior and antibacterial properties [14]. 

Tissues are formed by heterogeneous building blocks generating a complex hierarchy, from microscopic to macroscopic levels. Microscale hydrogels, i.e., microgels, are recognized as one of the methodologies that can create biomimetic engineered tissues [15]. Furthermore, additional surface functionalization with specific biomolecules could assist in cell adhesion, agglomeration and integration within the surrounding tissue [16]. 

Recently, our group has proposed the production of chitosan–metal ion spherical microgels for biomedical applications [17,18], using copper or zinc ions as bioactive components. Our previous results indicated facile preparation of spherical microgels using an electrohydrodynamic atomization process (electrospraying), and the biocompatibility of produced microgels was modulated by ion concentration. In this work, we propose the production of chitosan-based microgels simultaneously functionalized by copper (II) and zinc (II) ions, forming bimetallic-chitosan-complex microgels with a narrow size distribution and defined surface morphology. We also assessed the cytocompatibility of bimetallic chitosan microgels by varying the contents of Cu and Zn ions.

## 2. Materials and Methods

### 2.1. Materials

Chitosan with a deacetylation degree of 83.2% and viscosity of 293 mPa s (Chitoscience Chitosan 85/200; CHT83) and chitosan with a deacetylation degree of 96.9% and viscosity of 324 mPa s (Chitoscience Chitosan 95/200; CHT97) were purchased from Heppe Medical Chitosan GmbH (Halle (Saale), Germany). Acetic acid (99.8%) was purchased from Lach–Ner (Neratovice, Czech Republic), and sodium hydroxide (NaOH) was purchased from Honeywell (Seelze, Germany). Copper (II) acetate monohydrate (*M*_w_ = 199.65 g mol^−1^) and zinc (II) acetate dehydrate (*M*_w_ = 219.50 g mol^−1^), purchased from VWR Chemicals BDH (Leuven, Belgium), were used as precursors for Cu^2+^ and Zn^2+^ ions, respectively. Reagents used for washing of obtained chitosan-based microgels were 96% ethanol (Kefo, Ljubljana, Slovenia) and acetone (T.T.T. doo, Sveta Nedelja, Croatia). Sodium chloride, potassium chloride, sodium phosphate dibasic, and potassium phosphate monobasic were used for the preparation of phosphate-buffered saline solution (PBS, pH 7.4) and purchased from Gram-mol (Zagreb, Croatia). Lysozyme from chicken egg white (~70,000 U mg^−1^; Sigma-Aldrich, St. Louis, MO, USA) and sodium azide (VWR Chemicals BDH, Leuven, Belgium) were used in an enzyme-induced degradation of bimetallic chitosan microgels. All reagents were of analytic grade.

### 2.2. Preparation of Bimetallic-Chitosan-Complex Solutions

The Cu^2+^–Zn^2+^/chitosan complex solutions with different contents of metal ions were prepared by mixing a proper volume of 1 wt.% chitosan solution (in 0.5% *v*/*v* acetic acid) and a Zn^2+^-ion solution for 10 min (stirring speed was 350 rpm). After that, stirring was increased (700 rpm), followed by the addition of the Cu^2+^-ion solution. Mixing of the Cu^2+^–Zn^2+^/chitosan solution was continued for 50 min, after which a clear chitosan-based complex solution was obtained, with a final chitosan concentration of 0.88 wt.%. The total amounts of Cu^2+^ and Zn^2+^ ions in the bimetallic-chitosan-complex solution were added to respect the constant molar ratio (*n*(Cu^2+^) + *n*(Zn^2+^)):*n*(NH_2_) of 0.0915:1. Then, different molar ratios of copper/zinc ion-to-amino groups within the same complex solution were applied: *n*(Cu^2+^):*n*(NH_2_) = 0.0183:1 with *n*(Zn^2+^):*n*(NH_2_) = 0.0732:1 for sample Cu1-Zn4; *n*(Cu^2+^):*n*(NH_2_) = 0.0549:1 with *n*(Zn^2+^):*n*(NH_2_) = 0.0366:1 for sample Cu3-Zn2; and *n*(Cu^2+^):*n*(NH_2_) = 0.0915:1 for sample Cu5. Chitosan without metal ions was used as a control. Table 1 summarizes the Cu^2+^ and Zn^2+^ quantities needed for the preparation of bimetallic-chitosan-complex solutions, and the corresponding samples’ labelling.

### 2.3. Production of Bimetallic Chitosan Microgels

**Figure 1:**
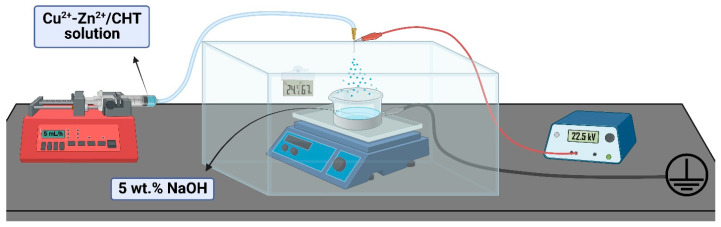
Schematic illustration of electrospraying setup. Created with Biorender.com.

In this work, the electrohydrodynamic atomization process (scheme shown in Figure 1) was used for the preparation of bimetallic chitosan (Cu^2+^–Zn^2+^/CHT) microgels. The required high voltage was determined by the formation of stable Taylor cone-jet mode at 22.5 kV for the given conditions in the process chamber (temperature of 24.1 ± 0.2 °C and relative humidity of 67 ± 5%). Parameters that were constant in all electrospraying processes were a 23-gauge needle, a bimetallic chitosan solution flow rate of 5 mL h^−1^, and the distance from needle-tip-to-collector of 10 cm. An NaOH solution (5 wt.%) was used as a gelation medium and collector of the formed microgels. The process procedure was followed as described in detail in our previous paper [18]. The obtained Cu^2+^–Zn^2+^/chitosan microgels were extensively washed with distilled water, 96% ethanol and acetone, and dried at room conditions. The same procedure was followed for the preparation of chitosan microgels (CHT83 and CHT97), which were used as controls.

### 2.4. Characterization Methods

The structural changes of chitosan were identified by FTIR spectroscopy using a Bruker Vertex 70 (Bruker Optics, Ettlingen, Germany) spectrometer equipped with a diamond crystal as an internal reflection element. Spectra were acquired in the wavenumber range from 4000 to 400 cm^−1^ with a resolution of 4 cm^−1^ and 32 scans at 20 °C.

To estimate the diameters of bimetallic chitosan microgels in the dry and wet states, all pictures gathered by light microscopes Olympus IX3 microscope (Olympus, Hamburg, Germany) with CellSens software for dry bimetallic chitosan microgels, and a BA200 binocular microscope (Motic Instruments, Barcelona, Spain) with Motic Images Plus 2.0 software for bimetallic chitosan microgels obtained after immersion, were processed by ImageJ 1.53e software. The diameter was estimated while assuming microgel sphericity of 1.

The swelling behavior of dry microgels was estimated as the volume ratio of microgels obtained after immersion in buffer solution and dry microgels, *V*_wet_/*V*_dry_ (µm^3^/µm^3^). 

The morphology of dry bimetallic chitosan microgels was investigated by scanning electron microscopy (SEM; Tescan Vega III Easyprobe, Brno-Kohoutovice, Czech Republic) with an electron beam energy of 10 keV. Before imaging, samples were sputter-coated with gold and palladium for 90 s.

### 2.5. Enzymatic Degradation

Enzymatic degradation of bimetallic chitosan microgels was investigated in phosphate-buffered saline solution (pH 7.4) supplemented with lysozyme (0.5 mg mL^−1^; LZ/PBS) and sodium azide (0.2 mg mL^−1^) at 37.5 ± 0.5 °C in an orbital shaker (50 rpm). Samples were weighed out to ~1 mg, and in vitro degradation was performed in 1.5 mL of medium for four weeks. Approx. 70% of fresh degradation medium was replaced every third day. After 4 weeks of incubation, degraded bimetallic chitosan microgels were investigated by light microscope to estimate the swelling capacity. Then, collected microgels were washed three times with distilled water, 96% ethanol and acetone, and left to dry at room conditions. The morphology of dry microgels obtained after degradation was investigated by SEM.

### 2.6. Cell Cytotoxicity Assay

The indirect MTT (3-(4,5-dimethylthiazol-2-yl)-2,5-diphenyltetrazolium bromide) assay was used as an indicator of cytotoxicity of prepared Cu^2+^–Zn^2+^/chitosan complexes against human embryonic kidney 293 cells (HEK293). The samples’ supernatant at a concentration of 0.5 mg mL^−1^ was used for cell feeding. Before assay, samples were sterilized by UV light for 30 min. The measurements were performed in triplicate.

HEK293 cells were cultured in Dulbecco’s modified Eagle’s medium with 4500 mg L^−1^ glucose (DMEM-high glucose; Capricorn Scientific) supplemented with 10% fetal bovine serum (FBS; Sigma-Aldrich, Burlington, MA, USA) and 1% penicillin/streptomycin (Sigma-Aldrich, Burlington, MA, USA). After reaching 80% confluence, cells were seeded into a 96-well plate (Sarsted) at a concentration of 5 × 10^4^ cells/200 µL of the medium and allowed to adhere overnight in a humidified incubator with 5% CO_2_ at 37 °C. Then, the medium was replaced by the samples’ supernatant, and cells were incubated for 72 h. Following the incubation period, the supernatants were removed, and cells were treated with 40 µL per well of MTT solubilized in cell medium at a concentration of 0.5 mg mL^−1^. After 3.5 h of incubation, 170 µL dimethyl sulfoxide (DMSO, Sigma-Aldrich, Burlington, MA, USA) was added to each well to dissolve the formazan crystals (15 min). The absorbance was measured at 560 nm using the microplate reader (Glomax-Multi, Promega). The cell viability was calculated as a percentage of untreated cells (negative control).

### 2.7. Statistical Analysis

The results are presented as mean values corrected by standard deviation. Data comparisons were carried out using the two-way analysis of variance (ANOVA) followed by Tukey post hoc test. A significant difference between groups is marked with an asterisk.

## 3. Results and Discussion

### 3.1. Structural Characterization

**Figure 2:**
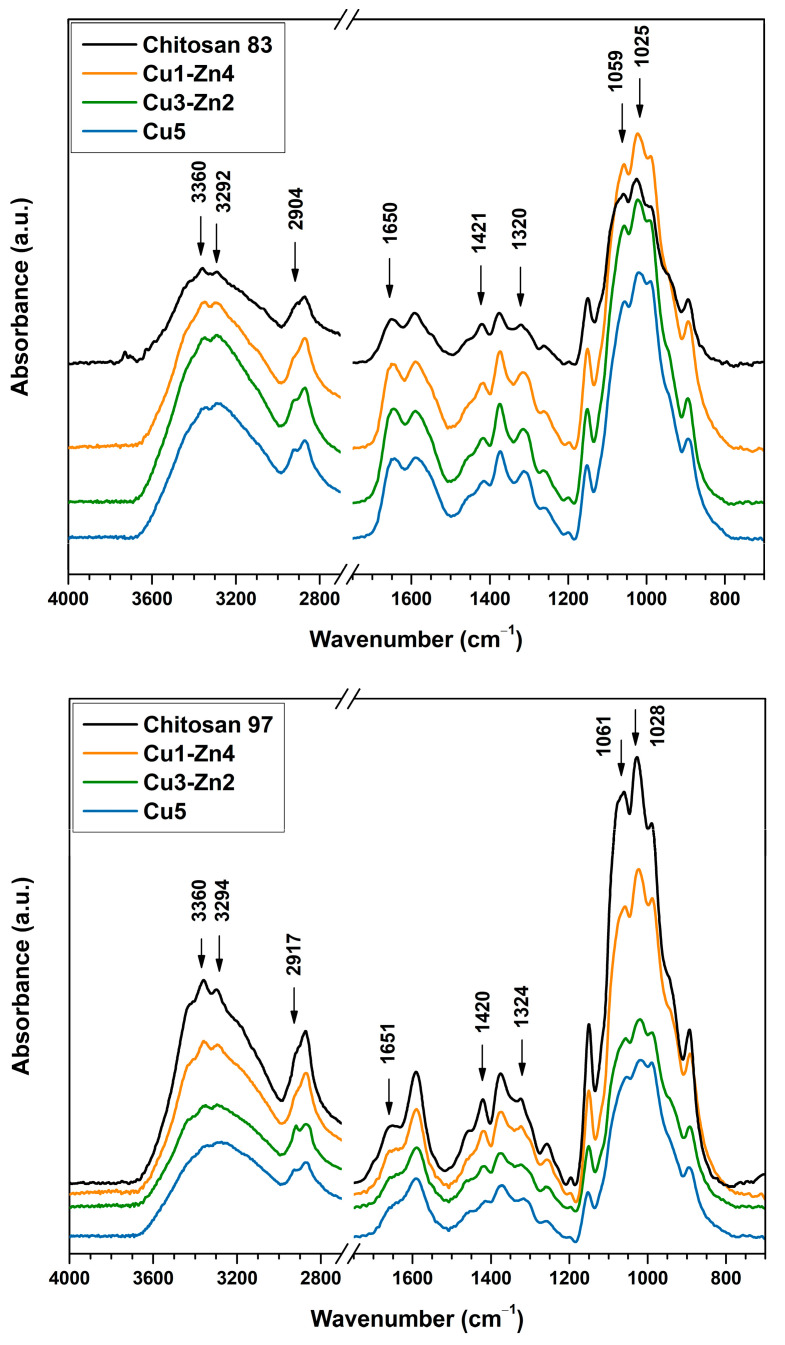
FTIR spectra of bimetallic chitosan dry microgels prepared with different contents of Cu^2+^ and Zn^2+^ ions.

The functionalization of chitosan with mono- and multivalent metal ions can be accomplished via simple complexation chemistry, where amino, amide and hydroxyl groups act as ligands in metal coordination. Such physical interactions can be sufficient to form strong physical crosslinks that significantly improve polymer’s mechanical properties [19]. The FTIR spectroscopy was used to evaluate the structure of dry bimetallic chitosan microgels prepared with different Cu^2+^ and Zn^2+^ content (Figure 2). The electrospraying of the chitosan solution without metal ions yielded precipitates of undefined sizes and shapes that were only used for structural characterization.

FTIR absorption bands characteristic for chitosan are summarized in Table S1, and the absorption bands with significant shifts in wavenumber are indicated in Figure 2. The addition of metal ions caused structural changes of chitosan, which are indicated by the band shifts to lower wavenumbers (except for the vibrations of the C–H bond). Dry microgels prepared from both chitosans (*DD*83 and *DD*97) showed significant changes for the same bond vibrations: an absorption band in the region of 3360–3292 cm^−1^ attributed to –NH_2_ and –OH stretching vibrations [20]; a band at 2904 cm^−1^ for chitosan *DD*83 and one at 2917 cm^−1^ for chitosan *DD*97 attributed to stretching of C–H bond in –CH_2_ group [12]; a band at ~1650 cm^−1^ assigned to C=O of the amide group; bands at ~1420 and ~1320 cm^−1^ attributed to bond vibrations of amide III [21]; and a region of 1059–1028 cm^−1^ attributed to stretching vibrations of the C–O bond of the glucosamine unit [14]. 

Chitosan *DD*97 systems showed a decrease in intensity of the C–O bond as the Cu^2+^ content increased, which could indicate more interactions between oxygen and copper [14]. Furthermore, a pronounced decrease in peak intensity at ~1590 cm^−1^ of *DD*97 bimetallic microgels was observed with higher Cu content. This absorption band is attributed to vibrations of the N–H bond in the primary amine, which indicates more involvement of –NH_2_ in metal coordination. The observed changes in the FTIR spectra of bimetallic chitosan systems are in agreement with previous studies performed on monometallic-chitosan complexes formed by copper (II), zinc (II) and iron (II) ions [10,14,22,23]. Those studies indicated that free hydroxyl and amino groups are involved in metal coordination, forming structures that can be described by two models, the “pendant” and “bridge” models [24]. Several types of [CuNH_2_(OH)_2_X] (X = H_2_O or –OH (chitosan)) complexes with “pendant” configuration were proposed [25], where copper is coordinated by the amino group, hydroxide ions and water molecules, depending on the pH of the aqueous solution. In addition, the “bridge” model is described by the tetrahedral coordination with amino groups of the same or different chitosan macromolecules.

All bimetallic systems were prepared at a constant molar ratio of metal ions-to-amino groups while varying the Cu^2+^ to Zn^2+^ ratio (see Table 1). Interestingly, the increase in the portion of Cu^2+^ ions, while decreasing that of Zn^2+^ ions, resulted in band shifts to lower wavenumbers, especially for bands attributed to hydroxyl and amino groups. Copper and zinc possess high affinity to primary amines, thereby forming Cu and Zn complexes. Copper has a stronger complexation capacity than zinc. Previously [9], we prepared monometallic chitosan complexes, and Cu–chitosan complexes had more stable structure at a lower concentration than Zn–chitosan-complex hydrogels. In the present study, similar behavior was observed on bimetallic chitosan microgels when more copper ions were added. It can be assumed that greater band shifts were the result of a stronger physical crosslink between chitosan and Cu^2+^ ions. The different coordination of Zn and Cu ions could be a reason for the different conformational changes of chitosan. While Cu ions have tetrahedral coordination, Zn ions have several coordination possibilities with glucosamine units and water molecules that could generate tetra-, penta- or hexa-coordination bonds [26], influencing the strength of Zn–chitosan interactions. 

### 3.2. Size Distribution of Dry Microgels

Recently, the use of microparticles (microgels) as building blocks for bottom-up tissue engineering strategies, 3D cellular disease models and reinforcement components in bioinks has extended microparticles’ applications in drug delivery and tissue engineering [27,28]. The design and engineering of microparticles are predominant in modulating biochemical-physical and architectural features that can induce a specific cellular response. Chitosan–metal complexes have shown potential as biologically active materials; however, those materials have been mainly prepared as films (coatings) or scaffolds with improved antibacterial and biological properties. Few studies have focused on preparing chitosan–metal complexes in the form of micro- or nanoparticles [22,29,30]. 

**Figure 3:**
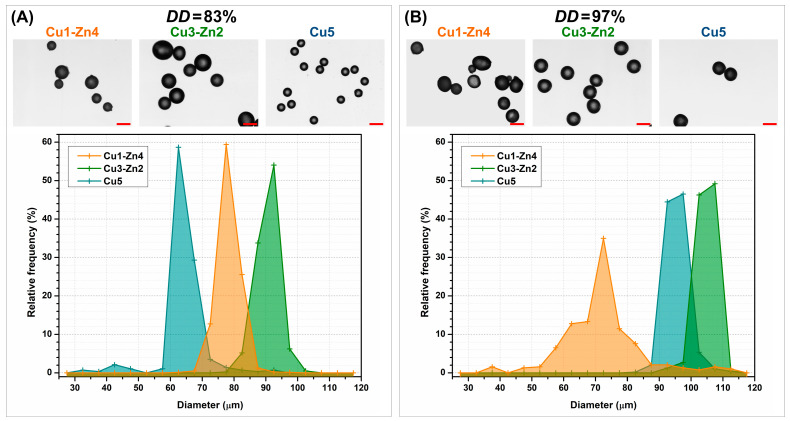
Particle size distribution and optical micrographs of dry bimetallic chitosan microgels produced from chitosan with (**A**) a deacetylation degree of 83% and (**B**) a deacetylation degree of 97%. The scale bar is 100 µm.

The size distribution of dry bimetallic chitosan microgels was estimated by light microscopy (Figure 3). Systems prepared from chitosan *DD*83 showed a narrow particle size distribution. The smallest size was observed for dry Cu5 microgels: an average size of 60–70 µm. The largest size was that of the Cu3-Zn2 microgels, which had an average size of 85–100 µm. Dry bimetallic chitosan microgels obtained from chitosan *DD*97 were larger in size in Cu5 (average size of 90–100 µm) and Cu3-Zn2 samples (average size of 100–110 µm) and had a narrow distribution. The Cu1-Zn4 system was characterized by a wider size distribution and an average size of 60–80 µm, similarly to Cu1-Zn4 *DD*83. Bimetallic-chitosan-complex solutions were prepared with the same metal ion-to-amino ratio, chitosan concentration and a similar molecular weight (declared by the manufacturer). A higher *DD* of chitosan required a slightly higher content of metal ions (chitosan *DD*97; see Table 1), which could have slightly changed the viscosity of complex solutions, and in the end, the size of the electrosprayed particles. It is important to note that due to the similar polymer solution viscosities, the same voltage was applied for all systems during the electrospraying process. Our previous work [18] has shown that monometallic-chitosan systems based on the chitosan with lower *DD* had somewhat higher viscosity than systems prepared with chitosan with higher *DD*. As a consequence, a stable Taylor cone-jet mode, which is necessary for the production of uniform size particles, was formed at different applied voltages. It can be assumed that voltage applied in this work was more favorable for the production of smaller particles with a narrower size distribution for *DD*83, consequently resulting in slightly bigger particles for *DD*97 systems. Nonetheless, the sizes of the produced bimetallic chitosan dry microgels were between 60 and 110 µm, independent of the chitosan deacetylation degree. 

Size and geometry are among the important physicochemical properties that must be in specific ranges for a microscale matrix to support anchorage-dependent cell expansion. Different particle sizes and shapes have been studied for this purpose, and several commercialized systems are characterized by a spherical shape and sizes between 90 and 400 µm [27]. Here, the functionalization of proposed chitosan-based microgels with Cu^2+^ and Zn^2+^ ions can produce spherical systems of such size and with a narrow size distribution by changing the metal-ion type and concentration.

### 3.3. Morphology of Dry Microgels

Other than a material’s surface chemistry and stiffness, which are key features for microcarrier design, surface topography can induce a specific cell response depending on the micro- and nanosized geometrical features.

**Figure 4:**
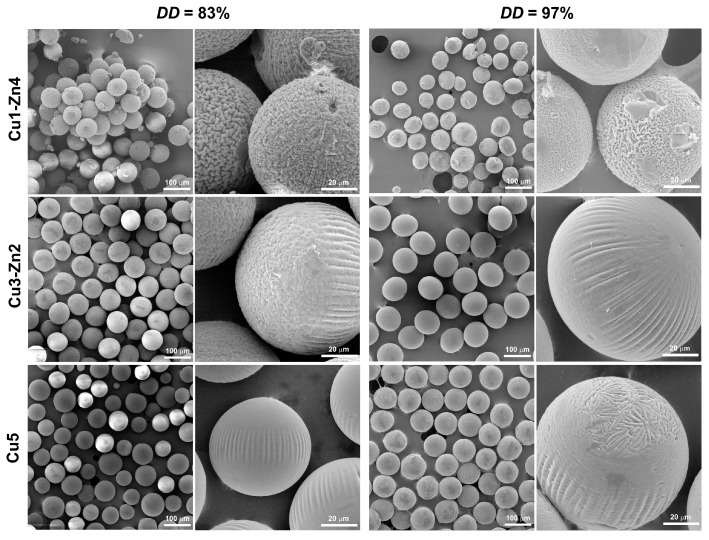
SEM micrographs of dry bimetallic chitosan microgels prepared from chitosan with different deacetylation degrees. The scale bars are 100 and 20 µm.

The influence of metal ions on surface morphology was observed on SEM micrographs of dry microgels (Figure 4). Gradual surface changes were observed on *DD*83 systems, from wrinkled morphology for Cu1-Zn *DD*83 to a smooth surface for Cu5 *DD*83. Previously, we have shown that copper (II) ions significantly influence the morphology of chitosan microparticles [18]. The evaluation of the size of as-prepared and dry microspheres indicated that the physical crosslinking through Cu^2+^ is responsible for surface changes. Wet chitosan microspheres prepared at a lower Cu^2+^ concentration had a larger size, and in dry state, they had a size similar to that of dry microspheres with higher *c*(Cu^2+^). We assumed that changes in surface morphology were the results of different amounts of Cu–chitosan crosslinks. In the present study, bimetallic chitosan microgels showed similar morphologies with higher Cu and lower Zn content. If Cu^2+^ ions can form stronger crosslinking in comparison to zinc ions, we can conclude that surface changes are governed by the metal that has more affinity towards chitosan functional groups. 

For *DD*97 bimetallic complexes, a smoother surface was observed, even for the Cu3-Zn2 *DD*97 dry microgel, which was similar to the topography of Cu5 *DD*83. It can be assumed that changes in surface topography are dictated by the content of Cu^2+^ ions. The microparticles produced at *w*(Cu^2+^) below 1.90% are characterized by a rough surface with wrinkles of a few microns, and increasing the copper (II) ion quantity above 3.12% led to a non-homogeneous surface with undefined regions, as observed for dry Cu5 *DD*97 dry microgel.

Micrometric topography is related to cellular adhesion, morphology and migration, and nanometric topography can stimulate cell proliferation, differentiation and alignment [31]. Here, we propose the use of a simple complexation chemistry between divalent metal ions and chitosan as a promising tool for engineering micro-sized systems with specific surface morphology. 

### 3.4. Swelling Property and Enzymatic Degradation

**Figure 5:**
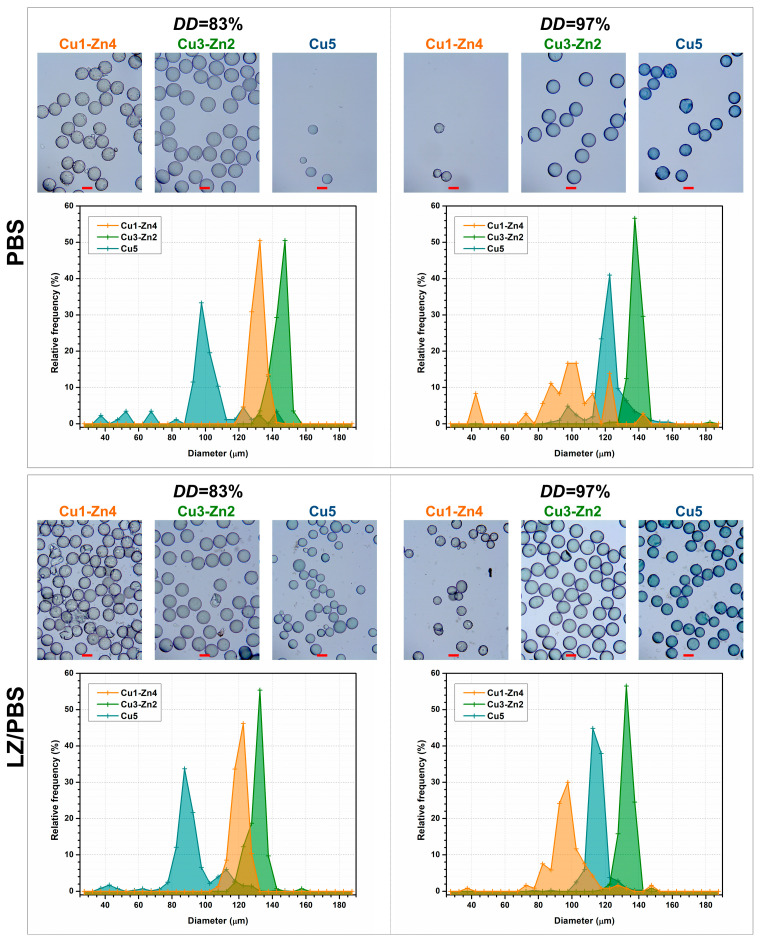
Microgel size distribution and optical micrographs of bimetallic chitosan microgels of chitosan with different deacetylation degrees (83 and 97%) obtained in phosphate-buffered saline solution (PBS, pH 7.4) after 24 h and after 4 weeks of incubation in lysozyme/PBS at 37 °C. The scale bar is 100 µm.

**Figure 6:**
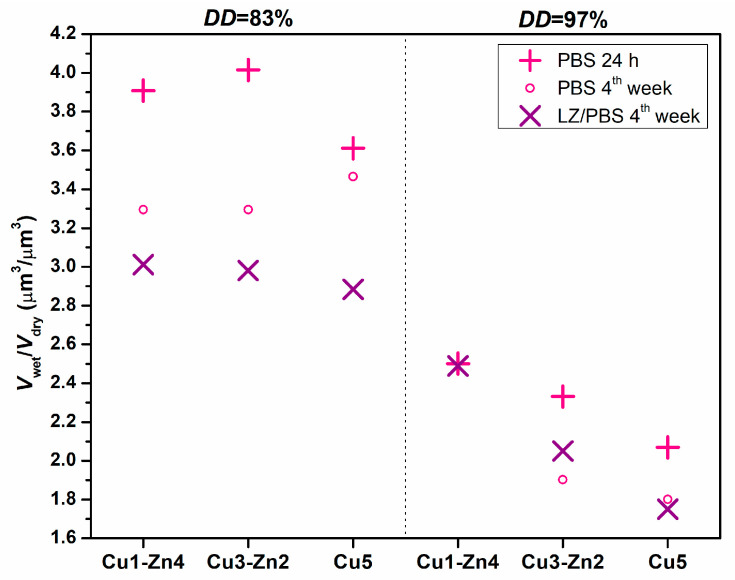
Volume ratio of bimetallic chitosan systems after immersion in phosphate-buffered saline solution (PBS, pH 7.4) or lysozyme/PBS solution, *V*_wet_, with respect to dry microgels, *V*_dry_. (Insufficient amount of Cu1-Zn4 *DD*97 sample at 4 weeks in PBS for analysis).

Swelling and biodegradation properties define the usefulness of chitosan-based materials in drug delivery and tissue engineering applications. The swelling behavior of bimetallic chitosan systems in phosphate-buffered saline solution (PBS), with and without lysozyme activity (LZ/PBS), was evaluated by analyzing the swollen particles (Figure 5) and wet-to-dry volume ratio (Figure 6).

The obtained microgels were transparent, spherical and blue at higher *w*(Cu^2+^). The size distribution was kept narrow (with exception for Cu1-Zn4 *DD*97) with larger size of microgels obtained after 24 h in PBS. A similar trend was observed on microgels after 4 weeks of degradation, which were characterized by a smaller size than those after 24 h in PBS.

Swelling capacity was estimated by the wet-to-dry volume ratio of microgels after particle immersion in buffer solutions (Figure 6). For chitosan *DD*83 microgels in PBS, the volume ratio between 3.6 and 4.0 was estimated, indicating good water absorption. On the contrary, chitosan *DD*97 microgels showed a lower absorption capacity with volume ratio values ranging from 2.0 to 2.5. The swelling ability of bimetallic chitosan microgels might give an insight into strength of the metal–chitosan crosslink. This effect was more evident for chitosan *DD*97 microgels, for which a slightly higher Cu^2+^ amount was added to respect the same metal-ion-to-amino molar ratio. Cu5 systems showed a lower volume ratio compared to Cu1-Zn4 samples, i.e., decreases of ~7.5% and ~17% for *DD*83 and *DD*97, respectively. We assume that more physical crosslinking between Cu^2+^ ions and chitosans’ functional groups might be responsible for lower absorption ability. A recent study [7] on mono- and bimetallic alginate hydrogels showed that the swelling property can be modulated by physical crosslinking using metal ions. The authors reported that Cu^2+^ and Fe^3+^ ions showed strong bonding interactions with alginate, forming tough and densely crosslinked monometallic hydrogels with a poor swelling property. This behavior was also maintained even in bimetallic hydrogels, where the second metal forms a weaker bond with the polymer matrix.

To clarify the notable difference in absorption ability between *DD*83 and *DD*97 bimetallic microgels, we evaluated the swelling capacities of chitosan samples without metal addition. Since electrospraying of chitosan solution without metal ions generated undefined precipitates, swelling was evaluated gravimetrically on solvent-casted films. After 24 h in PBS, chitosan *DD*97 film showed a lower water absorption capacity (2.79) with respect to the chitosan *DD*83 film (3.83). Lower swelling ability of chitosan with higher *DD* has already been reported in the literature [32], where more intermolecular bonds formed by more amino groups prevented water from entering into the polymer matrix. 

After 4 weeks of incubation in PBS and enzymatic medium (LZ/PBS), all bimetallic chitosan systems showed lower water absorption compared to 24 h of PBS immersion. Chitosan-based materials are hydrolyzed by enzymes lysozyme and chitinase, and this degradation depends on several parameters. The parameters that mainly control the degradation rate are the molecular weight and degree of deacetylation (molar percentage of glucosamine monomeric units) [33]. The viscosity, as an indication of molecular weight, was similar for chitosan *DD*83 and *DD*97, which means that the major impact on bimetallic microgels’ degradability would have the number of enzyme target sites, i.e., acetyl-glucosamine units [34]. Additionally, longer incubations in phosphate buffer showed certain weight loss of chitosan-based materials performed by chitosan dissolution [35].

**Figure 7:**
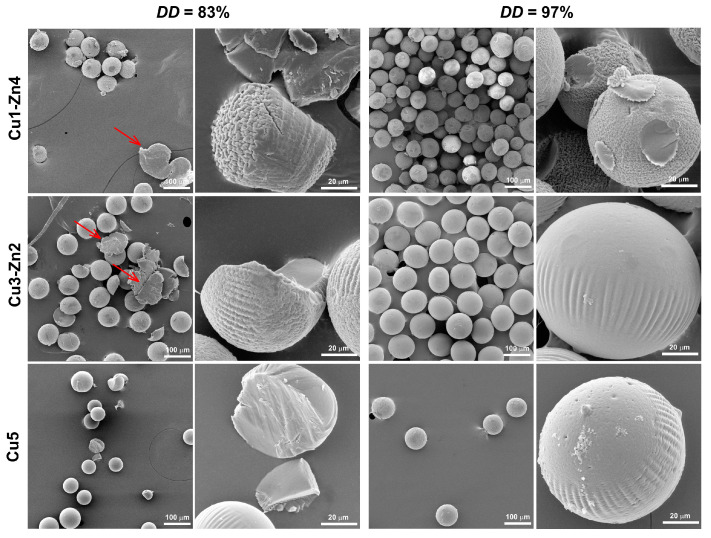
SEM micrographs of dry bimetallic chitosan microgels after 4 weeks of lysozyme degradation. The scale bars are 100 and 20 µm. Red arrows indicate microspheres’ disintegration.

Usually, enzymatic degradation of chitosan-based materials causes an increase in the material’s porosity, and consequently an increase in swelling capacity [36]. In this study, we performed accelerated enzymatic degradation at an extremely high lysozyme concentration, which should disintegrate the microgels, increase the porosity and consequently increase the water absorption. However, this was not observed for dry microgels after 4 weeks of enzymatic degradation—i.e., degraded samples were non-porous and spherical (Figure 7). A possible reason for decreased volume ratio could be a reduction in the size of microgels. Ren and coworkers [36] found that a decrease in the swelling ratio of compressed chitosan matrices is related to changes in sample dimension during enzymatic degradation. They proposed bulk erosion divided into two stages, were both swelling and degradation existed at the beginning of degradation. After reaching the maximum of swelling, the degradation process continues, leading to weight loss caused by thinning the sample. Furthermore, studies on enzymatic degradation of chitosan-based nanoparticles concluded that particle size reduction is a measure of in vitro degradation [37,38]. During 4 weeks of incubation, we noticed a reduction in the sample mass, ending with insufficient quantity for the evaluation of the size of dry degraded microgels. However, after 4 weeks of incubation in PBS and LZ/PBS, microgels showed lower swelling, which can indicate a smaller particle size. This was more evident for bimetallic microgels with *DD*83 incubated with lysozyme, as they had a decrease in volume ratio of up to 25% compared to swelling in PBS after 1 day. On the other hand, microgels prepared from chitosan *DD*97 showed a decrease in volume ratio of up to 16% with respect to the volume ratio after 1 day of PBS immersion. This observation could indicate a lower particle size reduction at a higher *DD*, as was previously shown in chitosan-based nanoparticles [37]. 

The surface morphology was maintained even after 4 weeks of lysozyme activity. However, bimetallic chitosan *DD*83 particles proved to be less stable, leading to particle disintegration with visible precipitates of non-defined shape (red arrows). Additionally, we observed less quantity after 4 weeks of enzymatic degradation for chitosan *DD*83 systems in comparison to chitosan *DD*97 systems. On the contrary, chitosan *DD*97 systems appeared more stable, again with maintained sphericity but without significant disintegration. The degradation via hydrolysis involves the reaction of weak bonds in polymer with water, and the degradation rate depends upon the polymer matrix’s permeability to water [39]. Chitosan with higher *DD* is more resistant to lysozyme degradation due to strong intermolecular interactions that decrease water permeation and the degradation rate.

### 3.5. Cell Cytotoxicity Assay

**Figure 8:**
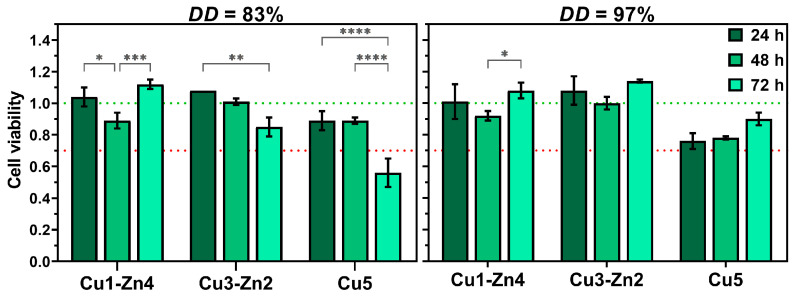
Cell viability of HEK293 cells cultured with material’s supernatant for 24, 48 and 72 h at 37 °C. Significant difference between groups: * *p* < 0.05, ** *p* < 0.01, *** *p* < 0.001, **** *p* < 0.0001.

Copper and zinc ions have proved their potential as antibacterial, angiogenic and osteogenic components of biomedical materials. In addition to antibacterial properties, copper is one of the most important metals for the human body, since it is involved in several physiological functions, from increasing the expression levels of pro-angiogenic and growth factors (VEGF or FGF-2) to regulating bone metabolism and turnover [40,41]. Zinc is an essential trace element which is needed for immune system functioning and bone development and plays a vital role in the wound healing process [13,14,41]. Despite positive effects on different cellular processes, the allowed concentration of copper is limited by the oxidative pathways of Cu^2+^ ions responsible for copper’s cytotoxicity. Hence, the cell cytotoxicity assay was performed to evaluate the toxicity of prepared bimetallic chitosan complex systems (Figure 8). 

After the first 24 h of incubation, cells showed good viability for all bimetallic complexes. Moreover, cell proliferation could be observed for bimetallic complexes containing more zinc (II) ions. The incubation during 72 h indicated a negative effect of copper on cell viability, especially for *DD*83 Cu5, where cell viability was decreased to ~50%. On the contrary, the *DD*97 Cu5 system showed ~76% cell viability after 24 h, without a significant difference between incubation times. The cytotoxic effect of *DD*83 Cu5 after 72 h could be associated with pronounced Cu^2+^ ion release in cell culture medium due to less free amino groups involved in metal chelation. Furthermore, systems with higher content of Zn^2+^ ions maintained good cell viability after 72 h, and even induced cell proliferation with respect to the systems where only copper ions were added. We can conclude that the main influence on cell viability was the concentration of Cu^2+^ ions in the proposed bimetallic chitosan complexes.

## 4. Conclusions

The physicochemical properties of chitosan-based microgels can be improved through the formation of physical interactions between chitosan’s amino and hydroxyl groups and therapeutic metal ions. In this work, bimetallic chitosan microgels were prepared by an electrohydrodynamic atomization process. FTIR spectra of the samples showed shifts in amino and hydroxyl group bands to the lower wavenumbers, indicating the formation of Cu and Zn complexes. Higher band shifts for complexes with more *w*(Cu^2+^) ions could indicate stronger physical crosslinks between chitosan and Cu^2+^ ions. Furthermore, dry microgels showed a narrow size distribution (with sizes ranging from 60 to 110 µm) and high sphericity, with surface morphology changing from wrinkled to smoother. The swelling capacity of dry bimetallic microgels decreased with increases in *w*(Cu^2+^) and deacetylation degree, likely due to more physical crosslinking between Cu^2+^ ions and chitosans’ functional groups. Additionally, all bimetallic chitosan microgels showed lower water absorption after four weeks of incubation in enzymatic medium (LZ/PBS) and PBS, which could indicate particle size reduction. The cytocompatibility of bimetallic chitosan complexes can be modulated by the content of copper (II) ions. These results demonstrate the potential of bimetallic chitosan microgels as microsized matrices for tissue engineering applications.

## Figures and Tables

**Table 1:** The weight fractions (*w*) of Cu^2+^ and Zn^2+^ ions in bimetallic-chitosan-complex solutions. *DD* represents the deacetylation degree of chitosan.

Sample		Cu1-Zn4	Cu3-Zn2	Cu5
*DD*83	*w*(Cu^2+^), %	0.62	1.84	3.03
	*w*(Zn^2+^), %	2.51	1.27	-
*DD*97	*w*(Cu^2+^), %	0.64	1.90	3.12
	*w*(Zn^2+^), %	2.58	1.31	-

## Data Availability

The data presented in this study are available upon reasonable request from the corresponding author.

## Supplementary Materials

The following supporting information can be downloaded at: https://www.mdpi.com/article/10.3390/polym15061480/s1, Table S1: FTIR absorption bands characteristic for chitosan in different bimetallic-chitosan dry microgels.

## References

[1] Xia H., Li X., Gao W., Fu X., Fang R.H., Zhang L., Zhang K. (2018). Tissue repair and regeneration with endogenous stem cells. Nat. Rev. Mater..

[2] Nikolova M.P., Chavali M.S. (2019). Recent advances in biomaterials for 3D scaffolds: A review. Bioact. Mater..

[3] Rial-Hermida M.I., Rey-Rico A., Blanco-Fernandez B., Carballo-Pedrares N., Byrne E.M., Mano J.F. (2021). Recent Progress on Polysaccharide-Based Hydrogels for Controlled Delivery of Therapeutic Biomolecules. ACS Biomater. Sci. Eng..

[4] Kurtuldu F., Kaňková H., Beltrán A.M., Liverani L., Galusek D., Boccaccini A.R. (2021). Anti-inflammatory and antibacterial activities of cerium-containing mesoporous bioactive glass nanoparticles for drug-free biomedical applications. Mater. Today Bio..

[5] Jungwirth U., Kowol C.R., Keppler B.K., Hartinger C.G., Berger W., Heffeter P., Hager S., Pape V.F., Pósa V., Montsch B. (2011). Anticancer Activity of Metal Complexes: Involvement of Redox Processes. Antioxid. Redox Signal..

[6] Ciriza J., Rodríguez-Romano A., Nogueroles I., Gallego-Ferrer G., Cabezuelo R.M., Pedraz J.L., Rico P. (2021). Borax-loaded injectable alginate hydrogels promote muscle regeneration in vivo after an injury. Mater. Sci. Eng. C.

[7] Shaheen A., Maswal M., Dar A.A. (2021). Synergistic effect of various metal ions on the mechanical, thixotropic, self-healing, swelling and water retention properties of bimetallic hydrogels of alginate. Colloids Surfaces A Physicochem. Eng. Asp..

[8] Yang R., Li G., Zhuang C., Yu P., Ye T., Zhang Y., Shang P., Huang J., Cai M., Wang L. (2021). Gradient bimetallic ion–based hydrogels for tissue microstructure reconstruction of tendon-to-bone insertion. Sci. Adv..

[9] Rogina A., Lončarević A., Antunović M., Marijanović I., Ivanković M., Ivanković H. (2019). Tuning physicochemical and biological properties of chitosan through complexation with transition metal ions. Int. J. Biol. Macromol..

[10] Gritsch L., Lovell C., Goldmann W.H., Boccaccini A.R. (2018). Fabrication and characterization of copper(II)-chitosan complexes as antibiotic-free antibacterial biomaterial. Carbohydr. Polym..

[11] Gu G., Erişen D.E., Yang K., Zhang B., Shen M., Zou J., Qi X., Chen S., Xu X. (2022). Antibacterial and anti-inflammatory activities of chitosan/copper complex coating on medical catheters: In vitro and in vivo. J. Biomed. Mater. Res. Part B Appl. Biomater..

[12] Brunel F., El Gueddari N.E., Moerschbacher B.M. (2013). Complexation of copper(II) with chitosan nanogels: Toward control of microbial growth. Carbohydr. Polym..

[13] Wang Y.-L., Zhou Y.-N., Li X.-Y., Huang J., Wahid F., Zhong C., Chu L.-Q. (2020). Continuous production of antibacterial carboxymethyl chitosan-zinc supramolecular hydrogel fiber using a double-syringe injection device. Int. J. Biol. Macromol..

[14] Mutlu N., Liverani L., Kurtuldu F., Galusek D., Boccaccini A.R. (2022). Zinc improves antibacterial, anti-inflammatory and cell motility activity of chitosan for wound healing applications. Int. J. Biol. Macromol..

[15] Santos L.F., Patrício S.G., Silva A.S., Mano J.F. (2022). Freestanding Magnetic Microtissues for Tissue Engineering Applications. Adv. Healthc. Mater..

[16] Correia C.R., Bjørge I.M., Zeng J., Matsusaki M., Mano J.F. (2019). Liquefied Microcapsules as Dual-Microcarriers for 3D+3D Bottom-Up Tissue Engineering. Adv. Healthc. Mater..

[17] Rogina A., Vidović D., Antunović M., Ivanković M., Ivanković H. (2020). Metal ion-assisted formation of porous chitosan-based microspheres for biomedical applications. Int. J. Polym. Mater. Polym. Biomater..

[18] Lončarević A., Ivanković M., Rogina A. (2021). Electrosprayed Chitosan–Copper Complex Microspheres with Uniform Size. Materials.

[19] Nie J., Wang Z., Hu Q. (2016). Chitosan Hydrogel Structure Modulated by Metal Ions. Sci. Rep..

[20] Li P., Feng Z., Yu Z., Chen Y., Li P., Yang Z., Li S., Jin S. (2019). Preparation of chitosan-Cu2+/NH3 physical hydrogel and its properties. Int. J. Biol. Macromol..

[21] Zhang Y., Wang D., Bai X., Xu J., Zhang J., Zhang G., Huang C., Liu W., Huang C., Xiong X. (2023). Microfluidic preparation of magnetic chitosan microsphere and its adsorption towards Congo red. J. Polym. Res..

[22] Perelshtein I., Ruderman E., Perkas N., Tzanov T., Beddow J., Joyce E., Mason T.J., Blanes M., Mollá K., Patlolla A. (2013). Chitosan and chitosan–ZnO-based complex nanoparticles: Formation, characterization, and antibacterial activity. J. Mater. Chem. B.

[23] Wang X., Du Y., Fan L., Liu H., Hu Y. (2005). Chitosan- metal complexes as antimicrobial agent: Synthesis, characterization and Structure-activity study. Polym. Bull..

[24] Ogawa K., Oka K., Yui T. (1993). X-ray study of chitosan-transition metal complexes. Chem. Mater..

[25] Rhazi M., Desbrières J., Tolaimate A., Rinaudo M., Vottero P., Alagui A. (2002). Contribution to the study of the complexation of copper by chitosan and oligomers. Polymer.

[26] Gomes J.R., Jorge M., Gomes P. (2014). Interaction of chitosan and chitin with Ni, Cu and Zn ions: A computational study. J. Chem. Thermodyn..

[27] Neto M., Oliveira M.B., Mano J.F. (2019). Microparticles in Contact with Cells: From Carriers to Multifunctional Tissue Modulators. Trends Biotechnol..

[28] Maciel M.M., Correia T.R., Henriques M., Mano J.F. (2022). Microparticles orchestrating cell fate in bottom-up approaches. Curr. Opin. Biotechnol..

[29] Yazdani M.R., Virolainen E., Conley K., Vahala R. (2017). Chitosan–Zinc(II) Complexes as a Bio-Sorbent for the Adsorptive Abatement of Phosphate: Mechanism of Complexation and Assessment of Adsorption Performance. Polymers.

[30] Wei D., Sun W., Qian W., Ye Y., Ma X. (2009). The synthesis of chitosan-based silver nanoparticles and their antibacterial activity. Carbohydr. Res..

[31] Sousa M.P., Arab-Tehrany E., Cleymand F., Mano J.F. (2019). Surface Micro- and Nanoengineering: Applications of Layer-by-Layer Technology as a Versatile Tool to Control Cellular Behavior. Small.

[32] Cao W., Jing D., Li J., Gong Y., Zhao N., Zhang X. (2005). Effects of the Degree of Deacetylation on the Physicochemical Properties and Schwann Cell Affinity of Chitosan Films. J. Biomater. Appl..

[33] Bagheri-Khoulenjani S., Taghizadeh S., Mirzadeh H. (2009). An investigation on the short-term biodegradability of chitosan with various molecular weights and degrees of deacetylation. Carbohydr. Polym..

[34] Tripathi A., Saravanan S., Pattnaik S., Moorthi A., Partridge N.C., Selvamurugan N. (2012). Bio-composite scaffolds containing chitosan/nano-hydroxyapatite/nano-copper–zinc for bone tissue engineering. Int. J. Biol. Macromol..

[35] Yang B., Li X., Shi S., Kong X., Guo G., Huang M., Luo F., Wei Y., Zhao X., Qian Z. (2010). Preparation and characterization of a novel chitosan scaffold. Carbohydr. Polym..

[36] Ren D., Yi H., Wang W., Ma X. (2005). The enzymatic degradation and swelling properties of chitosan matrices with different degrees of N-acetylation. Carbohydr. Res..

[37] Poth N., Seiffart V., Gross G., Menzel H., Dempwolf W. (2015). Biodegradable Chitosan Nanoparticle Coatings on Titanium for the Delivery of BMP-2. Biomolecules.

[38] Grenha A., Seijo B., Remuñán-López C. (2005). Microencapsulated chitosan nanoparticles for lung protein delivery. Eur. J. Pharm. Sci..

[39] Islam N., Dmour I., Taha M.O. (2019). Degradability of chitosan micro/nanoparticles for pulmonary drug delivery. Heliyon.

[40] Bosch-Rué E., Díez-Tercero L., Rodríguez-González R., Bosch-Canals B.M., Perez R.A. (2021). Assessing the potential role of copper and cobalt in stimulating angiogenesis for tissue regeneration. PLoS ONE.

[41] Glenske K., Donkiewicz P., Köwitsch A., Milosevic-Oljaca N., Rider P., Rofall S., Franke J., Jung O., Smeets R., Schnettler R. (2018). Applications of Metals for Bone Regeneration. Int. J. Mol. Sci..

