# Supplementary material for: Preparation and Properties of Bimetallic Chitosan Spherical Microgels
Source: Polymers (Basel). 2023 Mar 16;15(6):1480. doi: 10.3390/polym15061480 (PMC10057022; doi:10.3390/polym15061480)
Supplement: Supplementary file 1 [file polymers-15-01480-s001.zip › polymers-2264958-supplementary.pdf]

Supplementary Materials

Preparation and Properties of Bimetallic Chitosan Spherical Microgels

Andrea Lončarević [1,*], Karla Ostojić [2], Inga Urlić [2] and Anamarija Rogina [1,*]

[1] Faculty of Chemical Engineering and Technology, University of Zagreb, Trg Marka Marulića 19, HR-10000 Zagreb, Croatia
[2] Faculty of Science, University of Zagreb, Horvatovac 102a, HR-10000 Zagreb, Croatia;
* Correspondence: aloncarev@fkit.unizg.hr (A.L.); arogina@fkit.unizg.hr (A.R.)

Table S1. FTIR absorption bands characteristic for chitosan in different bimetallic-chitosan dry microgels.

Sample	Absorption band / cm^{-1}												
	–OH	$–NH_2$	C–H		C=O	N–H (amine)	Amide III				C–O–C, –COH		
**Chitosan 83**	3360	3292	2904	2871	1650	1591	1421	1375	1320	1260	1150	1059	1025
**Cu1-Zn4**	3348	3295	2928	2872	1649	1590	1416	1373	1315	1262	1150	1058	1023
**Cu3-Zn2**	3348	3295	2926	2870	1645	1590	1416	1375	1315	1264	1150	1058	1023
**Cu5**	3338	3286	2926	2872	1643	1590	1416	1373	1313	1264	1153	1056	1021
**Chitosan 97**	3361	3294	2917	2873	1651	1591	1420	1375	1324	1256	1150	1061	1028
**Cu1-Zn4**	3361	3295	2917	2872	1641	1590	1418	1373	1324	1256	1150	1058	1023
**Cu3-Zn2**	3350	3295	2917	2872	1641	1590	1416	1373	1324	1260	1150	1056	1019
**Cu5**	3340	3274	2928	2872	1641	1590	1414	1371	1317	1258	1153	1054	1019
